# Huntington's disease: of mice and men

**DOI:** 10.18632/oncotarget.15129

**Published:** 2017-02-06

**Authors:** Emma Yhnel

**Affiliations:** Neuroscience and Mental Health Research Institute, Psychological Medicine and Clinical Neurosciences, Cardiff University, Cardiff, UK

**Keywords:** Huntington's disease, mouse model, translation, cognitive training and ‘brain training’, Neuroscience

The genetic cause of Huntington's disease (HD) was identified as a trinucleotide expansion within the first exon of the *huntingtin* gene in 1993 [1]. As a result of this, numerous genetically modified animals have been created in an attempt to replicate the primary genetic cause of the human condition.

A large number of genetically modified animal models of HD are now available for use in research including large animal models such as non-human primates, sheep and pigs. However, by far the most commonly used genetically modified animal model of HD is the mouse. Knock-in mouse models in particular, contain the huntingtin mutation in the appropriate proteomic and genomic context to replicate the human condition. Mouse models of HD offer comparative ease of genetic manipulation and they are relatively inexpensive to maintain. However, the relative life span of a mouse in comparison to a human and the lack of gross structural neuroanatomical similarities is disadvantageous in modelling the human condition of HD.

A triad of motor, psychiatric and cognitive symptoms is described in the human condition of HD, with cognitive and psychiatric symptoms often occurring prior to the onset of motor symptoms [2]. While motor symptoms can be readily characterised in mouse models of HD utilising apparatus such as the rotarod, balance beam or staircase, cognitive and psychiatric symptoms are often more difficult to determine in mouse models of HD.

Operant testing apparatus allows the characterisation of cognitive dysfunction in mouse models of HD [3]. Although, in these mouse models it can often be difficult to tease out specific cognitive deficits from motor problems or general measures of apathy or motivation. However, cognitive problems are considerably burdensome for people living with HD, often from very early in the disease progression. Therefore, in determining how accurately a mouse model represents the human condition of HD, it is important to appropriately characterise cognitive changes in mouse models of the disease.

Cognitive changes are often characterised in a longitudinal manner, which requires repeated testing of animals in a battery of operant tasks [4]. Although different experimental designs can be used to minimise the practice effects of repeated cognitive testing, repeated cognitive testing in the same animals has been shown to modify HD related symptoms [5]. Furthermore, a cognitive training intervention utilising a five-choice serial reaction time task of attention, implemented early within the disease progression of HD, was shown to modify subsequent disease related behaviours in both wild type and HD mice [6]. Initial training, given at 4 months of age significantly changed subsequent behaviour 8 months later when the animals were tested in the same task. However, the possible transfer effects of such a cognitive training intervention into broader cognitive domains and tasks remain unclear. Thus, it future studies it may be necessary to include another task which tests similar cognitive functions to see if there is any generalisation to the benefits of the cognitive training intervention. Furthermore, modifications in response time were observed as a result of the cognitive training intervention, these are reported to be indicative of motor function. Therefore, it may be the case that cognitive training can influence the motor phenotype, although the possible mechanisms behind such an interaction remain unclear. Cognitive training studies in mouse models of HD have important implications for the longitudinal behavioural characterisation of HD mice, as repeatedly testing HD mice in cognitive tasks may well mask or modify the behavioural changes observed.

Pre-clinical studies suggest that cognitive training may provide some therapeutic benefit in HD. However, to maximise the beneficial effects of cognitive training in HD, it may be possible to use it in conjunction with other interventions in a combinatorial approach. For example, it could be combined with pharmacological interventions, transplantation therapies or non-pharmacological interventions such as exercise or changes to diet.

In conclusion, genetically modified mice were created in an attempt to model the human condition of HD, but the ultimate aim of this type of research is to translate and progress these findings into the patient clinic. Therefore, the next step is to translate the pre-clinical findings regarding cognitive training in mouse models of HD into the patient clinic. The first stage is to investigate the feasibility of such an intervention in this specific patient population, before determining if there are any functional benefits produced as a result of the intervention.
